# Information Entropy of Biometric Data in a Recurrent Neural Network with Low Connectivity

**DOI:** 10.3390/e27111125

**Published:** 2025-10-31

**Authors:** David Dominguez-Carreta, Mario González-Rodríguez, Francisco B. Rodriguez, Angel Sánchez, Rubem Erichsen

**Affiliations:** 1 Grupo de Neurocomputación Biológica, Dpto. de Ingeniería Informática, Escuela Politécnica Superior, Universidad Autónoma de Madrid, 28049 Madrid, Spain; f.rodriguez@uam.es; 2 SI2Lab, FICA, Universidad de las Américas, Quito 170124, Ecuador; mario.gonzalez.rodriguez@udla.edu.ec; 3 ETSII, Universidad Rey Juan Carlos, 28933 Madrid, Spain; angel.sanchez@urjc.es; 4 Instituto de Física, Universidade Federal do Rio Grande do Sul, Porto Alegre 91501-970, RS, Brazil; rubem@if.ufrgs.br

**Keywords:** neural networks, disordered systems, storage capacity, synaptic dilution, 87.18.Sn, 05.20.y, 75.10.Nr, 64.60.Cn

## Abstract

In this paper, we explore the storage capacity and maximal information content of a random recurrent neural network characterized by a very low connectivity. A specific set of patterns is embedded into the network according to the Hebb prescription, a fundamental principle in neural learning. We thoroughly examine how various properties of the network, such as its connectivity and the level of synaptic noise, influence its performance and information retention capabilities, which is evaluated through an entropy measure. Our theoretical analyses are complemented by extensive simulations, and the results are validated through comparisons with the retrieval of real biometric patterns, including retinal vessel maps and fingerprints. This comprehensive approach provides deeper insights into the functionality and limitations of finite-connectivity neural networks and their applicability to the retrieval of complex, structured patterns.

## 1. Introduction

Since the seminal work of Hopfield [1], Attractor Neural Network (ANN) remains as a permanent subject of investigation. With the recent development in the broad field of Artificial Intelligence and the plethora of its applications in modern life, this subject deserves renewed interest.

The Finite Connectivity Attractor Neural Network (FCANN) is an ANN where the neuron’s average connectivity is finite, which allows for a more realistic description of biological neural networks than the classical mean-field one [2]. The number of neurons and connectivities in biological systems are spread over a wide range. As extreme examples, C. elegans have around 300 neurons with average connectivity of around 10 [3,4], while in the human brain these numbers are in the order of $10^{11}$ and $10^{4}$, respectively. The FCANN model was introduced in [5], where an improved replica method was employed to investigate the thermodynamic properties of a network in which the patterns are stored according to a generalized Hebbian rule. In the present work we extend the formalism of [5] and develop the equations to explicitly evaluate the main observables.

Beyond the storage capacity, key properties of an ANN are the information content capacity and the ability to retrieve the information in different scenarios. The information capacity can be evaluated theoretically through entropy measurements [6]. The retrieval ability can be investigated through computer simulations or by applying to real problems. As a proof of concept the FCANN is used for retinal images retrieval. Digital Retinal Images for Vessel Extraction and Recognition is a research field within ophthalmology, which allows the early diagnosis of eye diseases such as diabetic retinopathy, glaucoma, and macular degeneration. Also, retina recognition is a biometric modality and technology that utilizes the unique patterns of blood vessels in the retina to identify individuals. This method is highly reliable due to the distinct and unchangeable nature of retinal patterns, which remain stable throughout the individual’s life [7].

Some main applications of retina recognition are the following ones:

(a) Security and Access Control: Retina images are used in high-security environments, such as military and government environments, to control access. Retina scanners are employed to secure restricted areas and ensure that only authorized personnel can enter.
(b) Healthcare: Medical identification systems use retina recognition to accurately match patients with their medical records. Retinal scans are also utilized in diagnosing and monitoring diseases like diabetes and hypertension, which affect retinal blood vessels.
(c) Banking systems: Financial institutions use retina recognition for secure authentication of transactions, ATMs, and online banking services to increase security and reduce fraud.

In recent years, the use of neural networks has emerged to automate and improve the accuracy of retina recognition [8]. Convolutional Neural Networks (CNNs) are particularly suitable for retina recognition due to their ability to extract relevant features from images through convolutional and pooling layers. It has been used for health monitoring and even inspired a particular CNN architecture [9]. Also other types of neural networks, such as multilayer feedforward perceptron, were considered to identify the individual to which a retina belongs. It is more precise than a human observer, automatic, efficient, and fast. However it cannot be used to reconstruct a complete noisy image [10]. In the present paper we propose to apply an extremely diluted Attractor Neural Network to recognize and retrieve a retinal structure from a noisy sample in a dataset.

The aim of this paper is to offer a deeper understanding of the FCANN, exploring features of its thermodynamics properties and its capabilities as an information storage device. It is organized as follows. Section 2 summarizes key findings and methods of related works. Section 3 offers a brief review of the replica method for finite connectivity networks, following the steps of [5]. In Section 4, a brief description of the calculation of information content is presented. In Section 5, we present an evaluation of the RS solution. Numerical simulations and retrieval of real retina patterns are described in Section 6. In Section 7 the results are presented. Some further remarks and conclusions are addressed in Section 8.

## 2. Related Works

The topology of the FCANN is that of the Erdös–Renyi network [11], consisting of *N* nodes, interacting with a finite neighborhood of nodes, randomly chosen, with average *c*. Finite connectivity disordered systems can be theoretically approached through different ways. One of them requires a large number (essentially infinite) of order parameters, like in [12]. The one that is adopted in the present work access the system’s properties by constructing the distribution of local fields acting on each site. See, e.g., ref. [13]. In the context of neural networks, it is used in ref. [5]. The technique has been successfully applied to disordered magnetic materials [14].

In previous works, the evaluation of information content has been crucial for understanding the dynamics of neural networks and spin models. Specifically, the information content of a fully connected three-state artificial neural network (ANN) was analyzed by ref. [15]. The study focuses on a three-state ANN, proposing a self-control model to explain low-activity patterns and examining an extremely diluted network. Furthermore, studies on threshold binary ANNs have demonstrated that these networks, when learning biased patterns, exhibit similar behaviors, with mutual information serving as a key measure of the network’s capacity as associative memory [16], where the authors explore the role of information measures in optimizing learning algorithms, contributing further to the understanding of neural network behavior. Finally, in ref. [6], it was shown that the mutual information expression as a function of macroscopic measures, such as the overlaps between patterns and neurons, can be used to derive new learning rules and even a complete original quartic (or biquadratic) ANN. This approach underscores the absolute relevance of using entropy information for ANNs, as it provides both a theoretical and practical framework for improving network performance.

Previous studies have also investigated extensions of Attractor Neural Networks to improve retrieval performance and storage capacity for both random and structured patterns. In ref. [17], a constructive heuristic and a genetic algorithm were proposed to optimize the assignment of retinal vessels and fingerprint patterns to ensembles of diluted attractor networks. By minimizing the similarity between pattern subsets using cosine, Jaccard, and Hamming metrics, the authors reduced cross–talk noise and increased the ensemble’s storage capacity, with validation on random, fingerprint, and retinal image datasets. The retrieval of structured patterns, specifically fingerprints, was also addressed in ref. [18], where a metric attractor network (MANN) was employed to exploit spatial correlations in the data. A theoretical framework linking retrieval performance to load ratio, connectivity, density, randomness, and a spatial correlation parameter was introduced, with good agreement between theory and experiments.

While the above approaches are rooted in statistical physics and theoretical neuroscience, recent trends in machine learning have shifted toward high-capacity architectures trained on large-scale image and biometric datasets. For instance, ref. [10] developed a retina-based identification system using feedforward neural networks trained via backpropagation, applying preprocessing, feature extraction, and classification stages. Their system, which used grayscale vascular segmentation from the DRIVE database, demonstrated the feasibility of automated personal identification using the retina as a biometric trait. More recent developments have focused on multimodal biometric systems that integrate multiple physiological characteristics. A representative example is the hybrid identification framework proposed by ref. [19], which combines convolutional neural networks (CNNs), Softmax, and Random Forest (RF) classifiers for the joint recognition of fingerprint, finger-vein, and face images. Their architecture applies K-means and DBSCAN algorithms for segmentation, exposure fusion for contrast enhancement, and CNNs as feature extractors, followed by classification through Softmax and RF layers. This line of research highlights the strong trend toward data-driven models for high-dimensional feature learning.

Complementary to CNN-based approaches, recurrent neural networks (RNNs) have also been explored for biometric and anomaly detection tasks. In ref. [20], the authors reviewed RNN applications in biometric authentication, expression recognition, and anomaly detection, emphasizing architectures such as Long Short-Term Memory (LSTM) and deep residual RNNs. These networks capture temporal dependencies in sequential biometric data such as gait, keystroke dynamics, or handwriting, achieving high recognition performance without requiring explicit spatial feature design. The review also underlines the versatility of RNNs for behavioral authentication and continuous monitoring applications.

In contrast to these modern machine learning approaches, characterized by dense connectivity, large parameter spaces, and supervised training on extensive datasets, the FCANN model developed in this work remains grounded in the information and statistical mechanics formulation of attractor networks. Our approach focuses on how information is represented, stored, and retrieved within a sparsely connected system, using entropy and mutual information as evaluation measures. In the present paper, we extend this analysis to an ANN with binary uniform patterns and very low connectivity. Despite the reduced connectivity, we find that entropy information remains essential for optimizing the system’s hyperparameters, namely, the temperature (external noise) and the learning ratio (internal noise). The search for an expression for entropy and the calculation of optimal parameters, aimed at maximizing the mutual information between neurons and the data, proves central to enhancing the overall efficiency of neural networks.

## 3. Methodology

**Figure 1:**
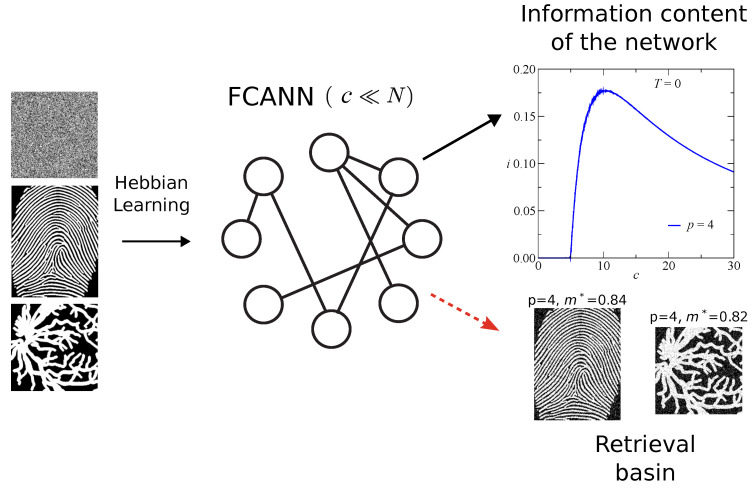
Finite Connectivity Attractor Neural Network (FCANN) schematic. Hebbian learning of random and biometric patterns (fingerprints and retinal vessels).

Figure 1 presents a schematic overview of the Finite Connectivity Attractor Neural Network (FCANN) framework employed in this work. The network is represented as a set of neurons connected through an Erdös–Rényi topology with finite average connectivity c≪N, emphasizing the sparse nature of the architecture compared to fully connected models. Patterns are embedded into the synaptic couplings following the Hebbian learning rule, with two distinct types of stored inputs: (i) random binary patterns, used for theoretical analysis and numerical simulations, and (ii) biometric patterns, specifically fingerprint images and retinal vessel maps, used to evaluate retrieval performance on real data. The diagram also illustrates the retrieval phase, in which the network reconstructs an original pattern from a noisy or partially degraded version, allowing the evaluation of the retrieval overlap *m* and the information content *i*. The retrieval basin of the system is illustrated in the figure for p=4 and $m^{*}\approx 0.8$, serving as a representative example of retrieval performance for a specific pattern load, and linking the conceptual schematic to the quantitative results presented in later sections.

### Model and Theoretical Development

The thermodynamics properties are accessed through the calculation of the partition function.

**Definition** **1.** *The system is composed by a set of formal neurons, represented by Ising variables $S_{i}=\pm 1$, i=1…N. A set of p=αc patterns $\left\{\xi_{i}^{\mu }\right\}$, μ=1…p, where α is a finite load parameter, is embedded in the couplings $J_{ij}$ through the Hebb’s rule*

(1) $$\begin{array}{c} J_{ij}=\frac{c_{ij}}{c}\sum_{\mu =1}^{p}\xi_{i}^{\mu }\xi_{j}^{\mu }\;, \end{array}$$

*where $c_{ij}$ is the probability that neurons i and j are connected. The $c_{ij}$ and $\xi_{i}^{\mu }$ are independent, identically distributed random variables satisfying, respectively, the distributions*

(2) $$\begin{array}{c} P\left(c_{ij}\right)=\frac{c}{N}\delta_{c_{ij},1}+\left(1-\frac{c}{N}\right)\delta_{c_{ij},0} \end{array}$$

*and*

(3) $$\begin{array}{c} P\left(\xi_{i}^{\mu }\right)=\frac{1}{2}\delta \left(\xi_{i}^{\mu }-1\right)+\frac{1}{2}\delta \left(\xi_{i}^{\mu }+1\right)\;, \end{array}$$

*where c is the average connectivity.*

**Definition** **2.** *The system’s Hamiltonian is given by*

(4) $$\begin{array}{c} H\left(\left\{S_{i}\right\}\right)=-\sum_{i,j<i}J_{ij}S_{i}S_{j}\;. \end{array}$$

*As usual, the thermodynamic properties are derived from the free-energy density*

(5) $$\begin{array}{c} f\left(\beta \right)=-\lim_{N\to \infty }\frac{1}{\beta N}{\langle \ln Z\rangle }_{\left\{c_{ij}\right\},\left\{\xi_{i}^{\mu }\right\}}\;, \end{array}$$

*where*

(6) $$\begin{array}{c} Z=\sum_{\left\{S_{i}\right\}}e^{-\beta H(\left\{S_{i}\right\})} \end{array}$$

*is the partition function.*

**Statement** **1.** The free-energy density can be written as

(7) $$\begin{array}{cc} f(\beta ) & =-\lim_{n\to 0}\frac{1}{\beta n}\mathrm{Extr}\{-\frac{c}{2}{\langle \sum_{SS^{\prime }}P_{\xi }\left(S\right)P_{\xi^{\prime }}\left(S^{\prime }\right)\left(e^{\frac{\beta }{c}\sum_{\mu }\xi^{\mu }\xi^{\prime \mu }\sum_{\alpha }S^{\alpha }S^{\prime \alpha }}-1\right)\rangle }_{\xi \xi^{\prime }}\\ & +{\langle \ln\sum_{S}\exp c{\langle \sum_{S^{\prime }}P_{\xi^{\prime }}\left(S^{\prime }\right)\left(e^{\frac{\beta }{c}\sum_{\mu }\xi^{\mu }\xi^{\prime \mu }\sum_{\alpha }S^{\alpha }S^{\prime \alpha }}-1\right)\rangle }_{\xi^{\prime }}\rangle }_{\xi }\}\;. \end{array}$$

**Proof.**  To deal with the averages in Equation (5), the replica method is applied,

(8) $$\begin{array}{c} f\left(\beta \right)=-\lim_{N\to \infty ,n\to 0}\frac{1}{\beta Nn}\ln{\langle Z^{n}\rangle }_{\left\{c_{ij}\right\},\left\{\xi_{i}^{\mu }\right\}}\;, \end{array}$$

where *n* is an integer. The replicated partition function reads

(9) $$\begin{array}{c} {\langle Z^{n}\rangle }_{\left\{c_{ij}\right\},\left\{\xi_{i}^{\mu }\right\}}={\langle \sum_{S^{1}\ldots S^{n}}\prod_{i,j<i}{\langle e^{\frac{\beta }{c}c_{ij}\sum_{\mu }\xi_{i}^{\mu }\xi_{j}^{\mu }\sum_{\alpha }S_{i}^{\alpha }S_{j}^{\alpha }}\rangle }_{\left\{c_{ij}\right\}}\rangle }_{\left\{\xi_{i}^{\mu }\right\}}\;, \end{array}$$

where $S^{\alpha }$ is a vector that represents the state of replica α. Averaging over $\left\{c_{ij}\right\}$, we have

(10) $$\begin{array}{c} {\langle Z^{n}\rangle }_{\left\{c_{ij}\right\},\left\{\xi_{i}^{\mu }\right\}}={\langle \sum_{S^{1}\ldots S^{n}}\prod_{i,j<i}\left[1+\frac{c}{N}\left(e^{\frac{\beta }{c}\sum_{\mu }\xi_{i}^{\mu }\xi_{j}^{\mu }\sum_{\alpha }S_{i}^{\alpha }S_{j}^{\alpha }}-1\right)\right]\rangle }_{\left\{\xi_{i}^{\mu }\right\}}\;. \end{array}$$

For N→∞, this can be written as

(11) $$\begin{array}{c} {\langle Z^{n}\rangle }_{\left\{c_{ij}\right\},\left\{\xi_{i}^{\mu }\right\}}={\langle \sum_{S^{1}\ldots S^{n}}\exp\left[\frac{c}{2N}\sum_{i,j\neq i}\left(e^{\frac{\beta }{c}\sum_{\mu }\xi_{i}^{\mu }\xi_{j}^{\mu }\sum_{\alpha }S_{i}^{\alpha }S_{j}^{\alpha }}-1\right)\right]\rangle }_{\left\{\xi_{i}^{\mu }\right\}}\;. \end{array}$$

To sum over the site variables, it is convenient to withdraw them from the inner exponential. To do this, we follow [5] and use the concept of sub-lattices. Sub-lattice $L_{\xi }$ is the set of sites *i* that $\xi_{i}=\xi$. Let us define $p_{\xi }\equiv N_{\xi }/N$, where $N_{\xi }$ is the number of sites belonging to $L_{\xi }$. With the sub-lattices, Equation (11) can be expressed as

(12) $$\begin{array}{cc} {\langle Z^{n}\rangle }_{\left\{c_{ij}\right\},\left\{\xi_{i}^{\mu }\right\}}=\langle \sum_{S^{1}\ldots S^{n}}\exp[\frac{c}{2N}\sum_{SS^{\prime }}\sum_{\xi \xi^{\prime }} & (e^{\frac{\beta }{c}\sum_{\mu }\xi^{\mu }\xi^{\prime \mu }\sum_{\alpha }S^{\alpha }S^{\prime \alpha }}\\ & -1)]\sum_{i\in L_{\xi }}\sum_{j\in L_{\xi^{\prime }}}\delta_{SS_{i}}\delta_{S^{\prime }S_{j}}\rangle_{\left\{\xi^{\mu }\right\}}\;. \end{array}$$

An explanation of the meaning of the vectors appearing in the above equation is needed. The replica labeled vectors $S^{1}\ldots S^{n}$ are *n N*-component vectors, meaning the whole spin state on each replica. The site labeled vector $S_{i}$ is an *n*-component vector which means the whole replica state in site *i*. Non-labeled vector S is a *n*-component vector which means the whole replica state of the one-site problem.

Contrary to the fully connected problem where, in the case of the replica symmetric solution, only the magnetization and the correlation between two replicas are the relevant order parameters, in finite connectivity problems higher-order correlations between replicas need to be taken in account [12]. This way, it is convenient to use the sub-lattice distribution of spin states,

(13) $$\begin{array}{c} P_{\xi }\left(S\right)=\frac{1}{N_{\xi }}\sum_{i\in L_{\xi }}\delta_{SS_{i}}\;, \end{array}$$

that is introduced in Equation (12) with the conjugated variables ${\hat{P}}_{\xi }\left(S\right)$:

(14) $$\begin{array}{cc} \langle Z^{n}\rangle =\sum_{S^{1}\ldots S^{n}} & \int \prod_{\xi S}\left[d{\hat{P}}_{\xi }\left(S\right)dP_{\xi }\left(S\right)\right]\exp[\sum_{\xi S}{\hat{P}}_{\xi }\left(S\right)\left(P_{\xi }\left(S\right)-\frac{1}{N_{\xi }}\sum_{i\in L_{\xi }}\delta_{SS_{i}}\right)\\ & +\frac{Nc}{2}{\langle \sum_{SS^{\prime }}P_{\xi }\left(S\right)P_{\xi^{\prime }}\left(S^{\prime }\right)\left(e^{\frac{\beta }{c}\sum_{\mu }\xi^{\mu }\xi^{\prime \mu }\sum_{\alpha }S^{\alpha }S^{\prime \alpha }}-1\right)\rangle }_{\xi \xi^{\prime }}]\;. \end{array}$$

Summing over the site variables and changing variables ${\hat{P}}_{\xi }\left(S\right)\to N_{\xi }\hat{P_{\xi }}\left(S\right)$, we have

(15) $$\begin{array}{cc} \langle Z^{n}\rangle \; & =\int \prod_{\xi S}\left[N_{\xi }d{\hat{P}}_{\xi }\left(S\right)dP_{\xi }\left(S\right)\right]\exp N[{\langle \sum_{S}{\hat{P}}_{\xi }\left(S\right)P_{\xi }\left(S\right)\rangle }_{\xi }\\ & +\frac{c}{2}{\langle \sum_{SS^{\prime }}P_{\xi }\left(S\right)P_{\xi^{\prime }}\left(S^{\prime }\right)\left(e^{\frac{\beta }{c}\sum_{\mu }\xi^{\mu }\xi^{\prime \mu }\sum_{\alpha }S^{\alpha }S^{\prime \alpha }}-1\right)\rangle }_{\xi \xi^{\prime }}\\ & +{\langle \ln\sum_{S}\exp\left(-{\hat{P}}_{\xi }\left(S\right)\right)\rangle }_{\xi }]\;. \end{array}$$

In the limit N→∞, the integral can be evaluated by the saddle-point method. The saddle point method, or steepest descent, is an asymptotic technique used to approximate integrals, especially those with a large parameter, like *N* in the equation above. The free-energy density, Equation (8), becomes

(16) $$\begin{array}{cc} f(\beta )\; & =-\lim_{n\to 0}\frac{1}{\beta n}\mathrm{Extr}\{{\langle \sum_{S}{\hat{P}}_{\xi }\left(S\right)P_{\xi }\left(S\right)\rangle }_{\xi }\\ & +\frac{c}{2}{\langle \sum_{SS^{\prime }}P_{\xi }\left(S\right)P_{\xi^{\prime }}\left(S^{\prime }\right)\left(e^{\frac{\beta }{c}\sum_{\mu }\xi^{\mu }\xi^{\prime \mu }\sum_{\alpha }S^{\alpha }S^{\prime \alpha }}-1\right)\rangle }_{\xi \xi^{\prime }}\\ & +{\langle \ln\sum_{S}\exp\left(-{\hat{P}}_{\xi }\left(S\right)\right)\rangle }_{\xi }\}\;, \end{array}$$

where Extr means that we take the extremum of the expression between braces relative to ${\hat{P}}_{\xi }\left(S\right)$ and $P_{\xi }\left(S\right)$, i.e., the variables need to satisfy the saddle-point equations

(17) $$\begin{array}{c} \frac{\partial f(\beta )}{\partial P_{\xi }\left(S\right)}=0 \end{array}$$

and

(18) $$\begin{array}{c} \frac{\partial f(\beta )}{\partial {\hat{P}}_{\xi }\left(S\right)}=0\;. \end{array}$$

Eliminating ${\hat{P}}_{\xi }\left(S\right)$, the remaining equation reads

(19) $$\begin{array}{c} P_{\xi }\left(S\right)=\frac{1}{N}\exp c\sum_{S^{\prime }}{\langle P_{\xi^{\prime }}\left(S^{\prime }\right)\left(e^{\frac{\beta }{c}\sum_{\mu }\xi^{\mu }\xi^{\prime \mu }\sum_{\alpha }S^{\alpha }S^{\prime \alpha }}-1\right)\rangle }_{\xi^{\prime }}\;, \end{array}$$

where $N=\sum_{S}P_{\xi }\left(S\right)$ is a normalization factor. Introducing Equation (19) in Equation (16), Equation (7) is obtained. □

As it will be explained below, all the thermodynamic observables can be obtained from the sub-lattices distributions of local fields $W_{\xi }\left(h\right)$, where

(20) $$\begin{array}{c} h_{i}=\sum_{j}J_{ij}S_{j} \end{array}$$

is the field acting on neuron *i* belonging to sub-lattice ξ due to the interaction with neighbor neurons.

**Statement** **2.** The distribution of the local fields in RS hypothesis is

(21) $$\begin{array}{cc} W_{\xi }\left(h\right) & =\sum_{k=0}^{\infty }p_{k}\langle \int \prod_{l=1}^{k}dh_{l}W_{\xi }\left(h_{l}\right)\\ & \times \delta \left[h-\sum_{l=1}^{k}\mathrm{arctanh}\left(\tanh\beta h_{l}\tanh\frac{\beta }{c}\sum_{\mu }\xi^{\mu }\xi_{l}^{\mu }\right)\right]\rangle_{\xi_{1}^{\prime }\ldots \xi_{k}^{\prime }}\;. \end{array}$$

**Proof.**  To solve Equation (19) we proceed with the RS *Ansatz* that assumes the invariance of $P_{\xi }\left(S\right)$ under permutation of replicas:

(22) $$\begin{array}{c} P_{\xi }\left(S\right)=\int dh\;W_{\xi }\left(h\right)\frac{e^{\beta h\sum_{\alpha }S^{\alpha }}}{{\left[2\cosh\beta h\right]}^{n}}\;, \end{array}$$

where $W_{\xi }\left(h\right)$ is the distribution of local field and is the main tool to evaluate all the relevant observables. Introducing the *Ansatz* in Equation (19) and expanding the exponential we have, unless it is for the normalization factor,

(23) $$\begin{array}{cc} P_{\xi }\left(S\right)=\sum_{k=0}^{\infty }p_{k}\langle \int & \prod_{l=1}^{k}\frac{dh_{l}W_{\xi }\left(h_{l}\right)}{{\left[2\cosh\beta h_{l}\right]}^{n}}\\ & \times \sum_{S_{1}^{\prime }\ldots S_{k}^{\prime }}\prod_{l=1}^{k}e^{\beta h_{l}\sum_{\alpha }S_{l}^{\alpha }+\frac{\beta }{c}\sum_{\mu }\xi^{\mu }\xi^{\prime \mu }\sum_{\alpha }S^{\alpha }S_{l}^{\alpha }}\rangle_{\xi_{1}^{\prime }\ldots \xi_{k}^{\prime }}\;, \end{array}$$

where $p_{k}=e^{-c}c^{k}/k!$. Summing over the site variables $S_{1}^{\prime }\ldots S_{k}^{\prime }$ in the right-hand side and rearranging terms, this becomes, in the limit n→0,

(24) $$\begin{array}{l} \int dh\;W_{\xi }\left(h\right)e^{\beta h\sum_{\alpha }S^{\alpha }}=\sum_{k=0}^{\infty }p_{k}\langle \int \prod_{l=1}^{k}dh_{l}W_{\xi }\left(h_{l}\right)\\ \;\;\times \exp\left[\left(\sum_{\alpha }\sigma^{\alpha }\right)\sum_{l=1}^{k}\mathrm{arctanh}\left(\tanh\beta h_{l}\tanh\frac{\beta }{c}\sum_{\mu }\xi^{\mu }\xi_{l}^{\mu }\right)\right]\rangle_{\xi_{1}^{\prime }\ldots \xi_{k}^{\prime }}\;. \end{array}$$

Solving for $W_{\xi }\left(h\right)$, we obtain

(25) $$\begin{array}{cc} W_{\xi }\left(h\right) & =\sum_{k=0}^{\infty }p_{k}\langle \int \prod_{l=1}^{k}dh_{l}W_{\xi }\left(h_{l}\right)\\ & \times \;\delta \left[h-\sum_{l=1}^{k}\mathrm{arctanh}\left(\tanh\beta h_{l}\tanh\frac{\beta }{c}\sum_{\mu }\xi^{\mu }\xi_{l}^{\mu }\right)\right]\rangle_{\xi_{1}^{\prime }\ldots \xi_{k}^{\prime }}\;. \end{array}$$

□

## 4. Network Performance: Entropy and Order Parameters

Knowing the distribution of local fields for all sub-lattices, we can evaluate the observables that allow to evaluate the performance of the network in handling information. The retrieval of a given pattern is signaled by a non-zero pattern overlap, in the limit N→∞

(26) $$\begin{array}{c} m=\frac{1}{N}\sum_{i=1}^{N}\xi_{i}{\langle S_{i}\rangle }_{T}\to {\langle \xi {\langle S\rangle }_{T}\rangle }_{\xi } \end{array}$$

where ${\langle \cdot \rangle }_{T}$ and ${\langle \cdot \rangle }_{\xi }$ mean thermal and disorder averages, respectively. This becomes, after applying the replica theory,

(27) $$\begin{array}{c} m={\langle \xi \int dh\;W_{\xi }\left(h\right)\tanh\left(\beta h\right)\rangle }_{\xi }\;. \end{array}$$

The spin glass order parameter

(28) $$\begin{array}{c} q=\frac{1}{N}\sum_{i=1}^{N}{\langle S_{i}\rangle }_{T}^{2}\to {\langle {\langle S\rangle }_{T}^{2}\rangle }_{\xi } \end{array}$$

becomes, in the replica symmetric solution,

(29) $$\begin{array}{c} q={\langle \int dh\;W_{\xi }\left(h\right)\tanh^{2}\left(\beta h\right)\rangle }_{\xi }\;. \end{array}$$

The first quantity upon which we may characterize the network performance is the storage capacity $\alpha_{max}$. It is defined as $\alpha_{max}=p_{max}/c$, where $p_{max}$ is the maximum number of patterns that still allow for a finite overlap *m*.

Beyond the storage capacity, the information content *i* is also a key quantity.

**Statement** **3.** The information content is given by

$$\begin{array}{c} i=\alpha I(S)\;, \end{array}$$

where

(30) $$\begin{array}{c} I\left(S\right)=\frac{1}{2}\left[(1+m)\ln(1+m)+(1-m)\ln(1-m)\right] \end{array}$$

is the mutual information that is a measure of the amount of information transmitted by a communication channel [6] and is an entropy measure, given by the difference between the output entropy S(S) and the entropy of the input signal S(S|ξ).

**Proof.**  According to the Shannon’s information theory [21], these entropies are given by

(31) $$\begin{array}{c} S\left(S\right)=-\sum_{S}P\left(S\right)\ln P\left(S\right) \end{array}$$

and

(32) $$\begin{array}{c} S\left(S;\xi \right)=-\sum_{S}P\left(S|\xi \right)\ln P\left(S|\xi \right)\;, \end{array}$$

where P(S) is the probability that a given neuron assumes the state *S*, and P(S|ξ) is the conditional probability that a given neuron assumes the state *S* given the pattern ξ. Averaging over disorder, we have

(33) $$\begin{array}{c} I\left(S\right)=-\sum_{S}P\left(S\right)\ln P\left(S\right)+{\langle \sum_{S}P\left(S|\xi \right)\ln P\left(S|\xi \right)\rangle }_{\xi }\;. \end{array}$$

Since our network is unbiased, P(S=1)=P(S=−1)=1/2. Then, Equation (31) becomes simply ln2. To evaluate the conditional entropy, Equation (32) and still considering that the patterns are unbiased, we just need for the conditional probabilities P(S|ξ) the quantities $P_{↑↑}$ and $P_{↑↓}$, i.e., the probabilities that the neurons are parallel or anti-parallel to the patterns. According to Equation (26), and using that $P_{↑↑}+P_{↑↓}=1$, we have

(34) $$\begin{array}{c} P_{↑↑}=\frac{1+m}{2} \end{array}$$

and

(35) $$\begin{array}{c} P_{↑↓}=\frac{1-m}{2}\;. \end{array}$$

From Equation (33),

(36) $$\begin{array}{c} I\left(S\right)=\ln2+P_{↑↑}\ln P_{↑↑}+P_{↑↓}\ln P_{↑↓} \end{array}$$

Replacing Equations (34) and (35) in Equation (36), we arrive at the expression in Equation (30), which depends only on *m* for any temperature because the patterns are unbiased. □

## 5. Stability of the RS Solution

In finite connectivity and disordered systems, the stability of the RS solution can be evaluated through the two-replica method [22,23], consisting of solving the saddle-point equations for two independent systems with the Hamiltonian

(37) $$\begin{array}{c} H\left(\left\{S_{i}\right\},\left\{S_{i}^{\prime }\right\}\right)=-\sum_{i,j<i}J_{ij}S_{i}S_{j}-\sum_{i,j<i}J_{ij}S_{i}^{\prime }S_{j}^{\prime }\;. \end{array}$$

The two systems share the same choice of random variables $J_{ij}$. The joint distribution of local fields $W_{\xi }\left(h,h^{\prime }\right)$ obeys the self-consistent equation

(38) $$\begin{array}{cc} W_{\xi }\left(h,h^{\prime }\right) & =\sum_{k=0}^{\infty }p_{k}\langle \int \prod_{l=1}^{k}dh_{l}\;dh_{l}^{\prime }\;W_{\xi }\left(h_{l},h_{l}^{\prime }\right)\\ & \times \;\delta \left[h-\sum_{l=1}^{k}\mathrm{arctanh}\left(\tanh\beta h_{l}\tanh\frac{\beta }{c}\sum_{\mu }\xi^{\mu }\xi_{l}^{\mu }\right)\right]\\ & \times \;\delta \left[h^{\prime }-\sum_{l=1}^{k}\mathrm{arctanh}\left(\tanh\beta h_{l}^{\prime }\tanh\frac{\beta }{c}\sum_{\mu }\xi^{\mu }\xi_{l}^{\mu }\right)\right]\rangle_{\xi_{1}^{\prime }\ldots \xi_{k}^{\prime }}\;. \end{array}$$

The overlap $q^{\prime }$, between two replicas, is given by

(39) $$\begin{array}{c} q^{\prime }={\langle \int dh\;dh^{\prime }\;W_{\xi }\left(h,h^{\prime }\right)\tanh\left(\beta h\right)\tanh\left(\beta h^{\prime }\right)\rangle }_{\xi }\;. \end{array}$$

The RS solution is stable if $W_{\xi }\left(h,h^{\prime }\right)$ is diagonal, i.e.,

(40) $$\begin{array}{c} W_{\xi }\left(h,h^{\prime }\right)=W_{\xi }\left(h\right)\delta \left(h-h^{\prime }\right)\;, \end{array}$$

which results in $q^{\prime }=q$ and is unstable otherwise.

## 6. Numerical Simulations

Simulations were realized to compare the model’s theory and experiment. A random network of *N* neurons with average connectivity *c* was created, with *p* embedded patterns according to Equation (1). To simulate a heat bath dynamics, each neuron was asynchronously updated according to

(41) $$\begin{array}{c} S_{i}\left(t+1\right)=\mathrm{sgn}\left[h_{i}\left(t\right)+z\right]\;, \end{array}$$

where sgn(x) returns the signal of *x*. The local field $h_{i}\left(t\right)$ is given by

(42) $$\begin{array}{c} h_{i}\left(t\right)=\sum_{j}J_{ij}S_{j}\left(t\right)\;, \end{array}$$

with the *j* summation running over all *i* neighbors and

(43) $$\begin{array}{c} z=T\arctan(u)\;, \end{array}$$

is Gaussian noise with u∈[−1,1] being an uniform random variable.

Beyond simulations with random input patterns, the algorithm above was applied to real input patterns consisting of retina images with N≈100,000 pixels.

## 7. Results

The main purpose in this paper is to resume the discussion of the FCANN model addressing some relevant questions not addressed before, like information capacity and RS stability.

All the observable are accessed through the calculation of $W_{\xi }\left(h\right)$ and $W_{\xi }\left(h,h^{\prime }\right)$ by using a population dynamic algorithm. There are $2^{p}$ sub-networks and, in principle, one local field distribution for each sub-network. Nevertheless, due to the reduction to a single neuron problem, there are only two distinct local fields distributions, one for the single neuron assuming the state +1, for a given pattern and other for the single neuron assuming the state −1, for a given pattern. Furthermore, if the patterns are not biased, like presently, the two distributions are mirrored. The population dynamic algorithm runs as follows, for each distribution: a population $\left\{h_{l}\right\}$ of fields is randomly created. We found that populations of N=100,000 fields and populations of N=500,000 produce similar results and we adopted N=100,000 throughout the paper. Then, for each iteration, (1) an integer *k* is chosen according to a Poisson distribution with average *c*; (2) *k* fields $h_{l}$ are randomly chosen from the population; (3) the summation in the Dirac’s δ-function in Equation (25) is calculated; and (4) a further local field is chosen and the result of the previous step is assigned to it. The procedure is repeated until convergence. The joint distribution Equation (38) is calculated similarly, except that two independently generated populations evolve with the same choice of randomness.

**Figure 2:**
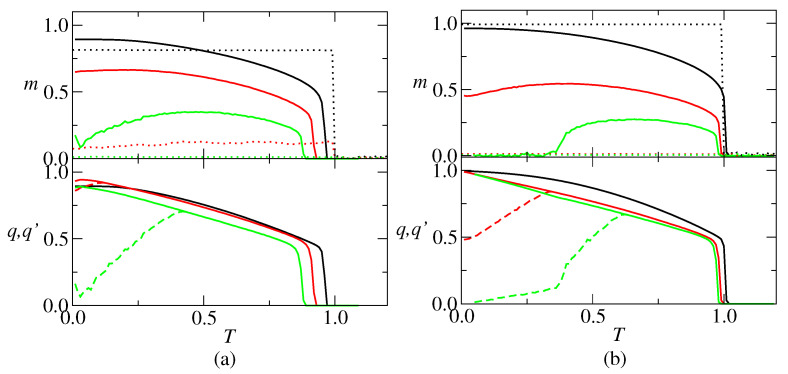
Retrieval overlap *m*, spin-glass parameter *q* (solid lines) and overlap between replicas $q^{\prime }$ (dashed lines) versus temperature *T*. (**a**) c=5, p=2 (black), p=3 (red) and p=4 (green); (**b**) c=20, p=5 (black), p=13 (red) and p=17 (green). The RS solution becomes unstable at $T<T_{AT}$, pointed out by the bifurcation between *q* and $q^{\prime }$. Dotted lines in the upper graphics represent simulation results for a network with N=100,000 neurons. In the lower graphics, the dashed lines represent the $q^{\prime }$.

In the RS solution, the thermodynamic behavior of the neural network is characterized by the retrieval overlap *m*, the spin glass parameter *q* and the overlap between replicas $q^{\prime }$. As an example of the outcome, plots of these parameters versus the temperature are presented in Figure 2, for representative connectivity values c=5 and c=20. The main distinction between them is that there is a re-entrant SG phase at low temperature for c=20, contrary to c=5. For c=5, there are two regimes: (i) p=2, there is a stable retrieval solution (R) with m>0 and $q=q^{\prime }>0$ for $0<T<T_{c}$ and a stable paramagnetic (PM) solution with $m=q=q^{\prime }=0$ for $T>T_{c}$, where $T_{c}$ is the critical temperature; (ii) p=3 and p=4, it appears an unstable retrieval solution (R’) with m>0 and $q>q^{\prime }>0$ for $0<T<T_{AT}$. For c=20, p=5, p=13 and p=17 are representative of three regimes, depending on α=p/c and the temperature, namely: (i) α=0.25, where there is a R solution for $T<T_{c}$ and a PM solution for $T>T_{c}$; (ii) α=0.65, where there is an unstable R solution for $T<T_{AT}$, a stable R solution for $T_{AT}<T<T_{c}$ and a PM solution for $T>T_{c}$; (iii) α=0.85, where there is a spin glass (SG) solution with m=0 and $q>q^{\prime }>0$ for $T<T_{SG}$, an unstable RS solution for $T_{SG}<T<T_{AT}$, a stable R solution for $T_{AT}<T<T_{c}$ and a PM solution for $T>T_{c}$. For comparison, the overlaps obtained from simulations on random networks with N=100,000 neurons, with average connectivity values c=5 and c=20, are also shown in Figure 2. The results show that, in the retrieval region, *m* is weakly dependent on *T*, decreasing abruptly to zero at T=1.0. Furthermore, it is worthy to remark that the simulated overlap decreases faster with the increase of *p*, as compared with the theoretical overlap. For example, for c=5 and p=3, the theoretical overlap is greater than 0.5 over a large range in temperature, while the simulated one is around 0.1. Contrary to the absolute value of the order parameters, theoretical and simulation results show a good agreement in which concerns the critical temperature for retrieval, with the agreement increasing with *c*.

**Figure 3:**
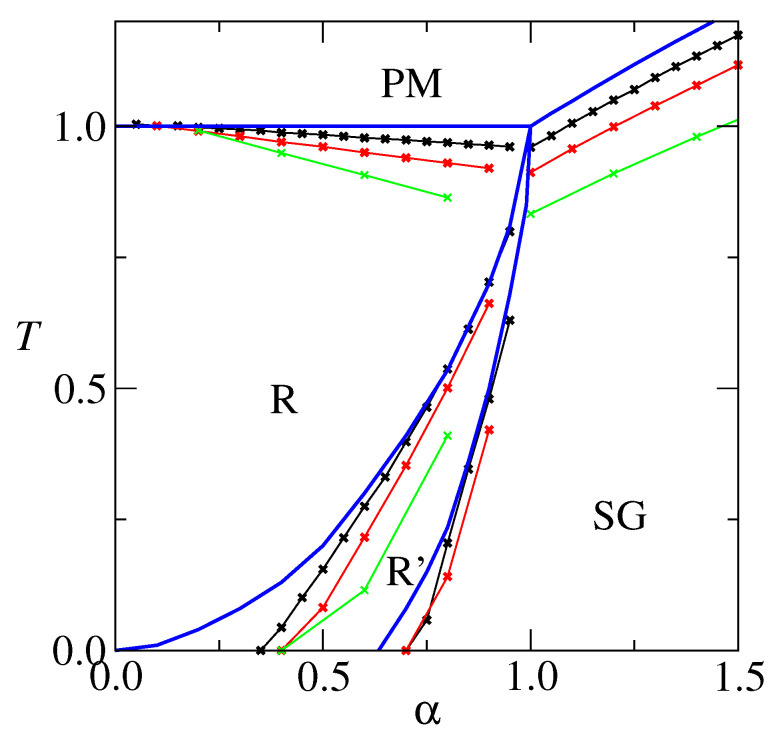
Phase diagram *T* versus α=p/c for c=20 (black) and c=10 (red) and c=5 (green). Symbols indicate the outcome of calculations, and lines are only to guide the eyes. For comparison, results for the extremely diluted c→∞ are also shown (blue), from ref. [24].

An overall picture of the model’s behavior can be achieved by drawing *T* versus α=p/c phase diagrams, which are presented, for c=5, c=10 and c=20, in Figure 3. For comparison, results for the extremely diluted c→∞ network [24] are also shown. The critical temperature $T_{c}=T_{c}\left(\alpha \right)$ signals the transition R-PM. As *c* increases, it approaches $T_{c}=1$, which is the c→∞ result. The AT line (from de Almeida-Thouless [25]) that signals the R-R’ transition displaces to the right, which means that the RS stable region increases as *c* decreases. In particular the finite *c* RS solution is stable at T=0, for a *c* dependent low α. This is in contrast to c→∞, where the RS solution is unstable for α>0. The freezing temperature $T_{f}=T_{f}\left(\alpha \right)$ that signals the SG-PM transition increases as *c* increases, approaching the limit $T_{f}=\sqrt{\alpha }$ for c→∞. The R’-SG transition deserves further attention. It is re-entrant, which may be credited to the RS solution instability. According to the full RSB Parisi’s scheme [26,27], the R (a ferromagnetic phase) to SG transition is a vertical line at α=1. We believe that, although difficult to calculate, this still applies to finite connectivity neural networks. It is worthy to remark that the re-entrance becomes less pronounced as *c* decreases and that it does not exist at all for c=5. Furthermore, the theoretical R’-SG transition for c=5 is close to α=1, which is the full RSB value. This suggests that low connectivity is capable of curing some of the pathologies associated with the RS solution.

**Figure 4:**
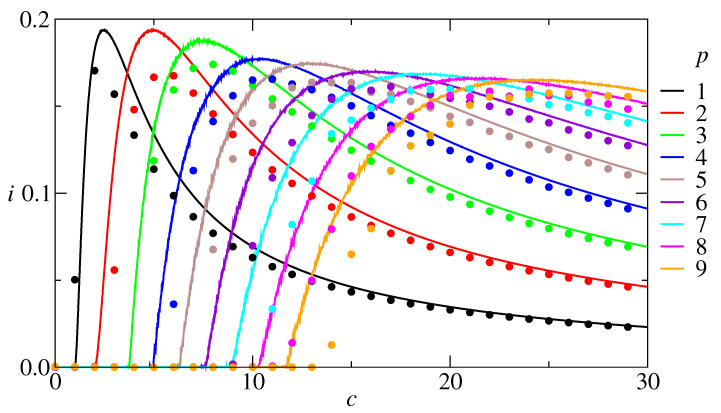
Information content as a function of the connectivity, at T=0, for several values of *p*, RS results. Dots: corresponding simulation results for a network with N= 100,000 neurons. The results show that for each number of patterns there is a connectivity that maximizes the information content. Furthermore, the maximal information content is obtained with a low number of patterns. This should be a valuable information for designers of artificial neural networks.

Since neural networks deal with information storage and retrieval, it is useful to investigate the relationship between the amount of information, the average connectivity, and the number of stored patterns. From a practical point of view, what is most promising: to built one dense network with a large *c* to store a large *p* or several less dense networks with a small *c* to store a small *p* in each one? A tentative answer is addressed in Figure 4, with the information content plotted as a function of the connectivity for *p* varying from 1 to 9, at zero temperature. The results show that *i* is a non-monotonic function of *c* and, consequently, for each *p* there is a *c* that maximizes *i*. The absolute maximum is $i_{\max}\approx 0.1936$, obtained for both p=1 and p=2 and then $i_{\max}$ slowly decreases for larger *p*. Curves for the information content versus connectivity were also evaluated through numerical simulations on a network with 100,000 units. The result, shown in Figure 4, shows a good agreement with the RS results, except that the theoretical results overestimate the simulation ones. We could enumerate five reasons for deviations from theoretical to simulations: (1) finite *N*, error around $1/\sqrt{N}∼0,3\%$; (2) discrete *c* in simulations versus continuous *c* in theory; (3) correlations between patterns for small *c*; (4) numerical errors in the evaluation of $W_{\xi }\left(h\right)$; and (5) RS hypothesis. All these errors combined can explain the discrepancies in Figure 2 and why the dots in Figure 4 are under the continuous curves.

**Figure 5:**
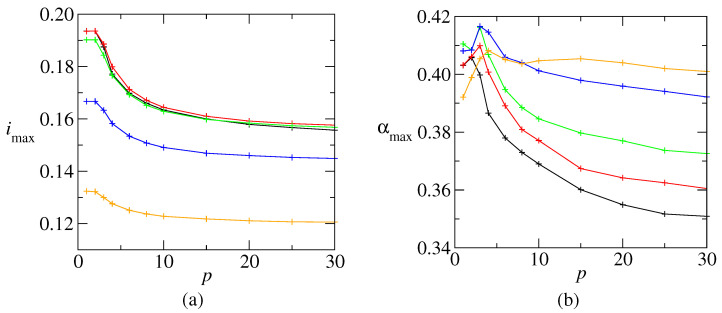
(**a**) Maximum information content $i_{\max}$ as a function of *p*, at T=0 (black), T=0.1 (red) T=0.2 (green), T=0.4 (blue) and T=0.6 (orange). (**b**) The corresponding $\alpha_{\max}=p/c_{\max}$, where $c_{\max}$ is the connectivity value that maximizes the information content, for each *p* value. The same color scheme as in (**a**). The lines are only to guide the eyes.

To clarify the tendency, Figure 5a shows $i_{\max}$ as a function of *p*. Initially, $i_{\max}$ decreases for p>2, but then it stabilizes for large *p*. The picture is qualitatively similar for larger temperatures. The figure also shows that $i_{\max}$ is not a monotonic decreasing function of the temperature. Instead, it slightly increases till T=0.1 before decreasing. This may be explained by the general argument that a low level of noise allows to avoid many spurious local minima. The load value $\alpha_{\max}$, corresponding to $i_{\max}$, is shown in Figure 5b. This value is close to 0.4 at low *p*, and then slowly decreases. It is worthy to remark that $\alpha_{\max}$ increases with *T* at low temperature which means that, for a given *p*, $i_{\max}$ appears at a lower *c*.

The simulation results presented so far refer to random input patterns. We may ask whether the theoretical predictions also hold for real-world visual data. To explore this, we employed binarized digital retina patterns derived from the DRIVE dataset [28], which contains high-resolution fundus images of human retinas. Each image was cropped to remove peripheral borders lacking structural information and then binarized to represent the main vascular structures as black–white patterns. The resulting images have a resolution of 316×316 pixels, corresponding to a total of 99,856 binary units per pattern.

The original mean activity of these binary retina patterns, measured as the proportion of white (active) pixels, was approximately 0.1021, indicating a strong bias toward sparsity. To produce input patterns more comparable to the random binary patterns used in the previous simulations, each image was morphologically dilated to increase the active proportion to approximately 0.5. This preprocessing step balances the input activity and facilitates a fair comparison between theoretical and empirical results.

**Figure 6:**
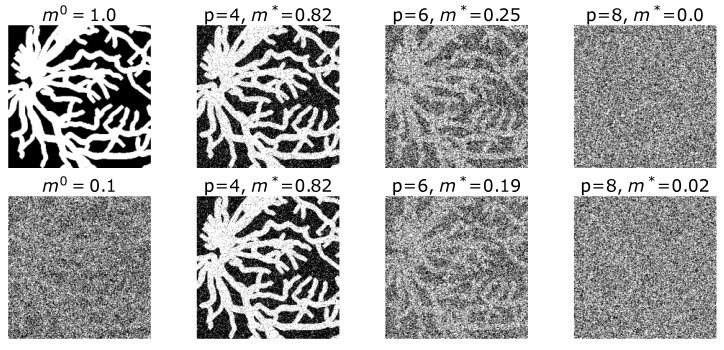
Retinal vessel patterns retrieval at T=0. The patterns are binarized images of retinas, consisting of N=316×316= 99,856 pixels and c=10.

The modified retina images were then used as real input patterns to evaluate the network’s retrieval ability at T=0 (noiseless retrieval), with a connectivity fixed at c=10. The results, presented in Figure 6, show that the fixed-point overlap decreases with the number of stored patterns, in agreement with Figure 2. Moreover, the overlap is only weakly affected by the level of initial noise, indicating that the retrieval state exhibits a large basin of attraction, demonstrating robustness for biometric structured input patterns.

A similar analysis was performed using binarized fingerprint images derived from the FVC2004: Third Fingerprint Verification Competition dataset [29] as real input patterns. The original images are in grayscale and were first gray-threshold binarized to emphasize ridge structures. The initial mean activity, measured as the proportion of white (active) pixels, was approximately 0.23, reflecting the intrinsic sparsity of fingerprint ridge patterns. To reduce bias and improve comparability with the random and retina-based patterns, the binarized images were morphologically dilated, yielding more balanced activity distributions while preserving the overall ridge topology.

The images were then cropped to retain the regions containing the most relevant fingerprint information, resulting in a final resolution of 340×263 pixels, corresponding to 89,420 binary units per pattern. As in the retina case, the network connectivity was set to c=10, and retrieval performance was evaluated at T=0 (noiseless retrieval).

**Figure 7:**
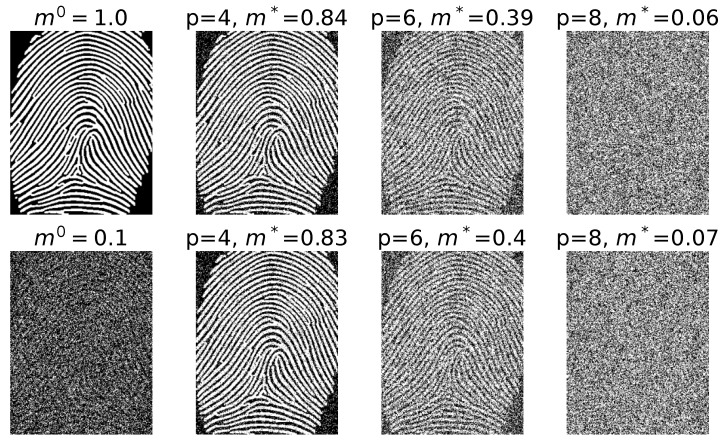
Fingerprint patterns retrieval at T=0. The patterns are binarized images of fingerprints, consisting of N=340×263= 89,420 pixels and c=10.

The results, shown in Figure 7, exhibit the same qualitative behavior observed with the retinal patterns: the fixed-point overlap $m^{*}$ decreases as the number of stored patterns *p* increases, while remaining relatively robust to variations in the initial noise level. These findings confirm that the FCANN model can successfully retrieve complex biometric patterns beyond retinal structures, further supporting the generality and robustness of the theoretical predictions when applied to structured, real-world data.

## 8. Discussion and Conclusions

Biological neural networks are extremely diluted, in the sense that each neuron interacts directly to a few fractions of the neuron population. Even then, the average connectivity amounts to several thousands of connections. Although there is no formal limit to the finite connectivity theory, in practice it is difficult to attain the biologic regime due to limitations in computation requirements: the computer time consumption is roughly proportional to the average connectivity. Nevertheless, the results presented in this paper may be significant to biological NN and, more than this, they may be relevant to applications in artificial neural networks, where the connectivity hardly compares to the biological realm.

Related to the previous work [5], we present the equations in an alternative form that allows to apply population dynamics and evaluate explicitly the order parameters as a function of the connectivity, learned patterns, and temperature. Also the information entropy was calculated, and we searched for the optimal information capacity as a function of the connectivity. Theoretical results were compared to simulations and biometric data.

A special attention was dedicated to the *T* versus α phase diagram. Using the two-replica method, the AT line for three representative *c* values was investigated. It was observed that for a connectivity as low as c=5, replica symmetry breaking effects, like the re-entrant behavior, are absent. This suggests that finite connectivity calculations is indeed an improvement, compared to the classical fully connected model. The RS transition from the unstable R to SG was also presented.

Numerical simulations with random and real input patterns were compared to the theory, showing a good agreement in predicting the R-PM transition, as well as in the information content. Nevertheless, there is a partial agreement in the prediction of the R’-SG transition. This subject deserves further investigation.

We keep thinking about biological and artificial neural networks. The results of this work also offer ideas about efficient information storage strategies. The energetic cost associated to the storage must be related both to the network unities (the neurons) as well as to the wiring (couplings). The results show that the maximal information “per coupling” is obtained for p=1 and 2, with low connectivity. If the wiring is most expensive than the unities themselves, this implies that low connectivity networks are more efficient to store information than densely connected networks. Meanwhile, the results also show that the maximal information decreases slowly with increasing *p*. This means that, if the relative wiring to unities cost is not too large, densely connected network could be an efficient strategy. Independently of the relative wiring and unities energy costs, an efficient neural network should operate in the range 0.35≲α≲0.4. It is worthy to remark that this range lies into the region where the RS solution is stable, and then it is a significant result.

Effects of thermal noise on information storage capacity were also investigated. The results show that a low level of noise (low *T*) are not harmful, and may even be beneficial, which means that sparse networks are robust against thermal noise.

## Data Availability

The original contributions presented in this study are included in the article. Further inquiries can be directed to the corresponding author.
